# A scoping review of music-based digital therapeutics for stress, anxiety, and depression

**DOI:** 10.3389/fnhum.2026.1602004

**Published:** 2026-03-13

**Authors:** Tara Venkatesan, Andrew M. Demetriou, Audrey Hempel, Daniel L. Bowling

**Affiliations:** 1 Universal Music Group, London, United Kingdom; 2 School of Advanced Study, University of London, London, United Kingdom; 3 Department of Intelligent Systems, Delft University of Technology, Delft, Netherlands; 4 Department of Psychiatry and Behavioral Sciences, Stanford School of Medicine, Stanford, CA, United States; 5 Center for Computer Research in Music and Acoustics (CCRMA), Stanford University, Stanford, CA, United States

**Keywords:** anxiety, depression, digital therapeutics, emotion regulation, mobile health, mood, music-based interventions, stress

## Abstract

Rising rates of stress, anxiety, and depression—fueled by rapid sociocultural and economic shifts, digital overexposure, and the lasting impact of COVID-19—are accelerating investment in scalable tools aimed at enhancing resilience and wellbeing. Music-based digital therapeutics (MDTs) hold promise given music’s unique ability to modulate core dimensions of health—affect, anxiety, and reward, as well as autonomic and social functioning—through a medium that is universal, intuitive, and increasingly accessible. To assess the current state of MDTs targeting stress, anxiety, and depression in adults, we conducted a scoping review using a modified Population, Intervention, Comparison, Outcome (PICO) keyword framework to structure Google search results. Twenty-two commercially available MDTs were identified for inclusion. We organize these MDTs into five principal categories based on underlying treatment strategies: (1) Preference-based music selection; (2) Affective Parameterization; (3) Affect Matching and Compensation; (4) Neural Entrainment; and (5) Biofeedback. We review general evidence supporting each strategy from music neuroscience and therapy research, as well as limited applied research testing specific MDTs. We conclude that, while general evidence supporting musical-based interventions for stress, anxiety, and depression is substantial, evidence for MDTs specifically is presently too limited to draw conclusions about real world effectiveness. Determining whether MDTs are likely to fulfill their potential will require increased focus on rigorous laboratory studies testing specific treatment strategies and randomized double-blind placebo-controlled trials conducted in ecologically valid settings. To support progress in this field, we make recommendations to support the sustainable development of MDTs as evidence-based tools to support mental health and wellbeing.

## Introduction

At the height of the COVID-19 pandemic, a survey of 23,000 + adults across 34 countries found evidence of a new peak in global stress levels. Roughly one in three (31%) respondents reported feeling overwhelmed by stress, one in four (27%) reported symptoms of major depression, and one in five (20%) said poor mental health prevented them from going to work—each on one or more occasions during the past year (Ipsos, 2022). Strikingly, two years later—and well past the height of the COVID-19 pandemic—the same survey found that these rates had roughly doubled, reaching 60% for overwhelming stress, 52% for major depressive symptoms, and 40% for occupational interference (Ipsos, 2024). Disproportionately affected groups include young adults, women, and low-income households (Ipsos, 2022, 2024). This is significant because, while acute stress is natural and often adaptive, chronic or overwhelming stress is harmful, heightening the risk of serious mental illness, cardiovascular disease, and other adverse health outcomes (Abdallah and Geha, 2017; Stress effects on the body, 2018; Clarke and Currie, 2009; Selye, 1978; Patriquin and Mathew, 2017).

In response to rising rates of stress, anxiety, and depression, conventional medicine offers a range of pharmacological and psychological treatments. While these first-line approaches constitute the foundation of modern psychiatric care and provide substantial benefits for many patients, extensive meta-analyses of randomized controlled trials report average summary effect sizes in the range of 0.3–0.4, indicating that many others derive limited benefit (Leichsenring et al., 2022). Beyond their modest overall efficacy, engagement with conventional treatments is limited by insurance gaps, cost, stigma, and medical distrust. For example, the treatment of anxiety disorders alone is estimated to account for approximately 2.1% of total healthcare costs in industrialized countries (Borson et al., 2019; Bugatti et al., 2023; Konnopka and König, 2020; Linardon et al., 2019; Ross et al., 2020), with an estimated 72% of those in need not receiving care (Alonso et al., 2018). This context has catalyzed the emergence of novel therapeutic approaches, including psychedelic medicines, brain stimulation, and music-based interventions (Cole et al., 2022; Carhart-Harris, 2018; Bowling, 2023; Gustavson et al., 2021).

### Music-based interventions

Music-based interventions (MBIs) are structured treatment programs that apply music to achieve therapeutic goals (Thaut and Hömberg, 2025). To date, they have developed most extensively within the field of *music therapy*. Music therapies—defined by the use of MBIs within a therapeutic relationship with a trained music therapist (Bruscia, 2014; Scope of Music Therapy Practice, 2015)—include active approaches focused on music making (e.g., singing, improvising, or composing alongside a trained therapist) and receptive approaches centered on music listening (e.g., to curated playlists or live performances, sometimes in combination with imagery or reflection).

Another MBI model is *music medicine*—a listening-based approach that typically involves daily, self-administered listening to a prescribed playlist (much like taking a pill; Bowling, 2023). Music medicine interventions do not necessarily require direct oversight from a music therapist, though such guidance is often involved during playlist development or initial prescription.

Recent meta-analyses summarizing hundreds of randomized controlled trials of MBIs—including both music therapy and music medicine—indicate a broad range of benefits, with medium-to-large effects on stress-related outcomes, anxiety, and depression (reviewed below). These findings provide a robust evidence base for the general application of MBIs to mental health and wellbeing, while also supporting their high acceptability, as individuals often find MBIs preferable, or even enjoyable, compared with other treatments (e.g., Bradt et al., 2015; Yuan et al., 2024).

Despite this evidence, the current impact of MBIs on stress, anxiety, and depression is strictly limited. One critical bottleneck is that existing treatment models largely rely on in-person care delivered by board-certified music therapists (e.g., MT-BC), which are relatively scarce. For example, there are only about 11,000 MT-BCs in the United States (American Music Therapy Association, 2021). Assuming an average caseload of 30 patients per week per therapist (Jackson, 2016), total treatment capacity can thus be estimated at less than 1% of potential demand—based on approx. 60 million U. S. adults experiencing clinically significant anxiety and/or depression in a given year (Kalin, 2020; National Institute of Mental Health NIMH, n.d.a; National Institute of Mental Health NIMH, n.d.b; Harvard Medical School, n.d.). Additional barriers to expanding MBI access include inconsistent recognition of music as an evidence-based intervention, limited integration into medical settings, and inadequate insurance coverage (Golden et al., 2022; Kern and Tague, 2017). Together, these factors constrain the scalability of current MBIs, limiting their potential population-level impact on mental health.

### Music-based digital therapeutics (MDTs)

Given the ubiquity of smartphones and the rapid growth of music streaming, the development of MDTs has emerged as a key approach for expanding access to MBIs. MDTs primarily follow the music medicine model, using self-administered, structured listening sessions delivered via smartphone to address barriers to MBI access. Proposed benefits of MDTs include reaching populations that lack access to music therapists, providing music-based treatment on-demand, and lowering costs (e.g., $119 per hour for in-person music therapy sessions versus $5–$20 monthly for digital applications; American Music Therapy Association, 2021). While these proposed benefits are plausible, they assume that MDTs confer benefits comparable to those of traditional MBIs—an assumption that has not been thoroughly investigated.

One reason to suspect that MDT benefits may be less than those of, e.g., music therapy, is the relative reduction of social factors that shape treatment effects. For example, the alliance that can develop between therapist and client can meaningfully contribute to treatment outcomes, e.g., by fostering trust, belief, and social connection (Itskovich et al., 2022). Yet the formation of such an alliance is not guaranteed, as it depends on the interpersonal dynamics of individual therapist–client dyads (Renner et al., 2012). Perhaps reflecting this variability, studies comparing traditional music therapy and music medicine (i.e., MBIs with and without therapist involvement) have generally found comparable effects (Bradt et al., 2015; Bradt et al., 2016; de Witte et al., 2022), and in some cases, stronger effects for music medicine (Tang et al., 2020; see also Tsoi et al., 2018).

While these findings are encouraging for MDTs and other forms of music medicine, it remains reasonable to assume that non–music-specific effects—particularly social and relational ones—enhance the effects of musical treatment in ways that are fundamentally limited by digital delivery. This implies that MDTs—or any approach to expanding the impact of MBIs by reducing reliance on the direct session-level involvement of music therapists—must focus on maximizing the contribution of music-specific effects. Importantly, such effects, and the MDTs that seek to deploy them are not mutually exclusive with therapist involvement. Hybrid models that combine MDTs with music-therapist oversight may integrate the strengths of both modalities, maintaining key social and relational factors while simultaneously providing a basis for scaling access and impact.

## Effects of music on stress, anxiety, and depression

At the broadest level, the evidence that music counteracts stress, anxiety, and depression comes from the fact that relaxation, mood modulation, and enjoyment consistently rank among the top reasons that people listen to music, a finding which has been replicated across multiple international surveys (Cook et al., 2019; Thoma et al., 2012; Uhlig et al., 2013; Vidas et al., 2021; Fink et al., 2021; Lonsdale and North, 2011). Likewise, meta-analyses of MBIs that follow both music therapy and music medicine models indicate that these self-reported reasons extend to experimental effects in sub-clinical and clinical contexts.

### Stress

Stress refers to a set of natural responses to environmental challenges mediated by neural circuits that link perception and cognition to highly conserved sympathetic and hypothalamic–pituitary–adrenal (HPA) axis systems. Acute stress facilitates adaptive responding by increasing cortical arousal, the supply of oxygen and glucose to the muscles, heart, and brain, and suppressing digestion and acute inflammation. But chronic stress is detrimental to health, precipitating disorders like generalized anxiety and major depression, either of which can feedback on sympathetic and HPA axis systems to further elevate stress (Stephens and Wand, 2012; Southwick et al., 2005; Tafet and Nemeroff, 2016). Assessing the impact of MBIs on stress physiology, a 2020 meta-analysis examining 61 randomized controlled trials (*N* = 3,188) found significant anti-stress effects on heart rate (*d* = −0.46), blood pressure (*d* = −0.34), and stress-related hormones (e.g., cortisol; *d* = −0.35), with comparable efficacy between traditional music therapy and music medicine approaches (de Witte et al., 2020). A subsequent meta-analysis from the same group, focusing on randomized controlled trials of music therapy, and averaging across physiological and psychological stress-related outcomes, found a larger effect (*d* = −0.72; 47 RCTs, *N* = 2,747), which they argued may have been related to benefits from the involvement of trained music therapists (de Witte et al., 2022).

### Anxiety

Anxiety refers to a disproportionate sense of fear or worry about distal threats and may be associated with restlessness, trouble concentrating, irritability, and sleep disturbances (Barlow, 2002; Craske and Stein, 2016; Grupe and Nitschke, 2013). Neurobiologically, anxiety is largely rooted in functional imbalance within the brain’s *negative affect* network, especially nodes in the medical prefrontal cortex (mPFC), anterior cingulate cortex (ACC), amygdala, bed nucleus of the stria terminalis, insula, and hypothalamus (Akiki et al., 2025; Martin et al., 2009; Rauch et al., 2003; Sylvester et al., 2012). Assessing the impact of MBIs on anxiety, a 2021 meta-analysis examining 32 controlled trials (*N* = 1,924) found a small-to-moderate anxiolytic effect of music therapy (SMD = –0.36; Lu et al., 2021). With greater relevance to MDTs, a 2018 meta-analysis of 81 randomized controlled studies (N > 6,000) found that listening to music before, during, or after surgery, offered by a music therapist or a researcher but typically listened to alone over headphones, had a moderate-to-large effect on anxiety (mean difference [MD] = −0.69; Kühlmann et al., 2018). Focusing on music medicine specifically, a 2023 meta-analysis of 21 (mostly) randomized controlled trials (*N* ≈ 1,800) found a large effect on anxiety (*d* = −0.77; Harney et al., 2023).

### Depression

Depression is primarily characterized by low mood and/or anhedonia and may be associated with changes in appetite/weight, sleep, fatigue, cognitive function, psychomotor function, self-reproach, or in severe cases, suicidality (Malhi and Mann, 2018; Zimmerman et al., 2008). The neural bases of depression overlap with stress and anxiety, including nodes in the mPFC, ACC, orbitofrontal cortex (OFC), amygdala, insula, nucleus accumbens (NAc), and hypothalamus, among other regions (Williams, 2016; Tozzi et al., 2024; Drevets et al., 2008; Keedwell et al., 2005; Russo and Nestler, 2013). Assessing the impact of MBIs on depression, a 2020 meta-analysis of 55 studies (*N* = 3,984) indicated stronger effects for music medicine (SMD = –1.33) versus music therapy (SMD = –0.66; Tang et al., 2020). However, an earlier meta-analysis of 9 music therapy studies (9 controlled trials; *N* = 411) also found a large effect on depression (SMD = –0.98; Aalbers et al., 2017).

Together, these findings provide substantial evidence that MBIs—including both music therapy and music medicine—can reduce stress, anxiety, and depressive mood, with moderate-to-large effect sizes. While it may be assumed that such effects are likely to transfer to MDTs, we emphasize that none of the above meta-analyses include specific analyses of MDTs, which remain a relatively novel approach to music-based intervention.

### Underlying mechanisms

At a psychological level, music’s effects on stress, anxiety, and depression have been attributed to several factors, including emotional regulation (e.g., Ginsberg et al., 2022), positive reminiscence (e.g., Ashida, 2000), and distraction (e.g., Aitken et al., 2002; Radstaak et al., 2014).

At a neurobiological level, substantial evidence indicates that music directly modulates core affective and reward circuitry in the brain. A foundational discovery in music neuroscience is that pleasurable music listening stimulates classical brain reward systems, including the mesolimbic dopamine pathway and associated mu-opioid signaling (Alluri et al., 2012; Garza-Villarreal et al., 2015; Salimpoor et al., 2013; Zatorre, 2015; Mallik et al., 2017; Mas-Herrero et al., 2023). Functional connectivity analyses further show that pleasurable music enhances temporal coupling between key reward nodes (e.g., nucleus accumbens) and brain regions involved is stress and autonomic regulation (e.g., hypothalamus; Menon and Levitin, 2005). This coupling also extends to a broader affective network—including the amygdala, insula, orbitofrontal cortex, medial prefrontal cortex, and anterior cingulate cortex—regions also reflected in coordinate-based meta-analyses of music-evoked brain activity (Koelsch, 2020) and strongly implicated in psychiatric neuropathology (Williams, 2016; Akiki et al., 2025).

A second major advance concerns the neural basis of auditory-motor entrainment to rhythm. Musical rhythms spontaneously entrain neural oscillations across a distributed sensory-motor network encompassing auditory, parietal, motor, and premotor cortices, as well as basal ganglia, thalamus, and cerebellum (Chen et al., 2008; Grahn and Rowe, 2009). Notably, this network is activated even in the absence of movement, suggesting that rhythm engages the brain even when a person is unwilling or unable to move. The degree to which a rhythm engages this network—captured by the construct of musical “groove”—is closely linked to both musical pleasure (Janata and Parsons, 2013) and nucleus accumbens activity (Matthews et al., 2020). With respect to stress, anxiety, and depression, these findings suggest that high-groove rhythms may dynamically engage attention and sensorimotor systems, potentially interrupting maladaptive cognitive patterns (e.g., anxious perseveration or depressive rumination) while concurrently activating reward-related circuitry (see Bowling, 2023).

Finally, emerging evidence for song- and music-selective neural responses in the human superior temporal gyrus (STG)—existing in parallel to speech-selective responses—suggests that music engages a specialized component of auditory neurobiology (Norman-Haignere et al., 2015, 2022). Given music’s exceptional capacity to modulate emotion, this component appears to be tightly integrated with affective and reward networks. This suggests that sensitivity to musical affect and reward may be relatively preserved in individuals in which these functions are otherwise compromised (Leipold et al., 2023; Abrams et al., 2026). Together, these findings suggest that music’s ability to alleviate stress, anxiety, and depression stems from enjoyment-driven neuromodulation of core affective and reward networks in the brain.

## Emerging music-based digital therapeutics

MDTs are under rapid development and are already being commercialized with advertised benefits for health and wellbeing, specifically targeting the regulation of stress, anxiety, and mood. While the general evidence supporting music’s therapeutic effects is strong, the degree to which modern commercial MDTs reproduce or enhance these effects (as many claim) remains unclear. In this section, we review the approaches to musical ‘treatment’ undertaken by these MDTs, focusing on their connection to the evidence reviewed above, as well as application-specific peer-reviewed research toward validation of their health benefits.

## Methods

### Search strategy

We conducted a scoping review of music-based digital therapeutics (MDTs) using a structured around a modified Population, Intervention, Comparison, Outcome (PICO) framework implemented via Google’s search engine (Eriksen and Frandsen, 2018). This approach was necessary because MDTs represent a nascent and rapidly evolving application domain that is not adequately captured by conventional database-driven systematic review methods.

Specifically, keyword searches of bibliographic databases such as PubMed and Web of Science proved ineffective, as studies evaluating interventions later incorporated into specific MDTs rarely reference the MDT by name—or even identify the intervention as “music-based” or a “digital therapeutic”—in titles, keywords, or sometimes the text itself. This reflects the fact that many MDT applications were developed only after the underlying scientific work was published. Accordingly, a web-based strategy was required to identify commercially available MDT products and their associated documentation.

In the modified PICO framework, intervention search terms included *“music,” “music intervention,” “music medicine,”* or *“music-based”* combined with technology-related terms such as *“mobile application,” “smartphone,” “tablet,” “mHealth,” “app,” “computer,” “mobile-based,”* and *“technology-based.”* Outcome terms included *“anxiety,” “calm,” “stress,” “depression,” “mood,” “emotion,”* and *“negative affect.”* The population term was fixed as *“adult.”* Comparison terms were excluded, as most MDTs have been developed within consumer- or business-facing models and do not explicitly define comparator conditions.

### Inclusion and exclusion criteria

For conceptual clarity, inclusion was restricted to MDTs that primarily employed receptive approaches, thereby isolating listening as the principal therapeutic ingredient rather than motor, expressive, or interpersonal engagement. MDTs incorporating music as one component of broader health or wellness programs were included even when music was not the primary focus, as such hybrid approaches constitute a substantial portion of current digital health practice and are therefore directly relevant. MDTs identified through the search that, upon further review, did not employ music (e.g., relied on noise-based stimuli) were excluded.

To focus on MDTs positioned within the evidence-based digital health sector, inclusion further required that associated documentation referenced one or more of the following terms: *“science,” “science-backed,” “science-based,” “scientifically validated,”* or *“scientifically proven.”* Although this criterion is inherently imprecise—being influenced by marketing language and not synonymous with true scientific validation—it served as a pragmatic means of constraining the search to discrete MDT products rather than diffuse content such as individual videos or playlists (e.g., on YouTube or Spotify).

For practical reasons, inclusion was also limited to applications that were described in English and associated with an active, publicly accessible product and/or web presence at the time of review. Applications consisting solely of informal media content, as well as those lacking an identifiable product or platform, were excluded.

### Search timing and data sources

The search was conducted in January 2025 and reflects a snapshot of the MDT landscape at that time. In accordance with scoping review methodology—which emphasizes transparent, time-bounded mapping of an evolving evidence landscape rather than exhaustive or continuously updated inclusion—applications released after this date were not included, and applications that later became defunct were not retrospectively excluded (Tricco et al., 2018). Given the rapid pace of commercial development in digital health, this approach provides a transparent and methodologically appropriate basis for mapping the field.

**Figure 1:**
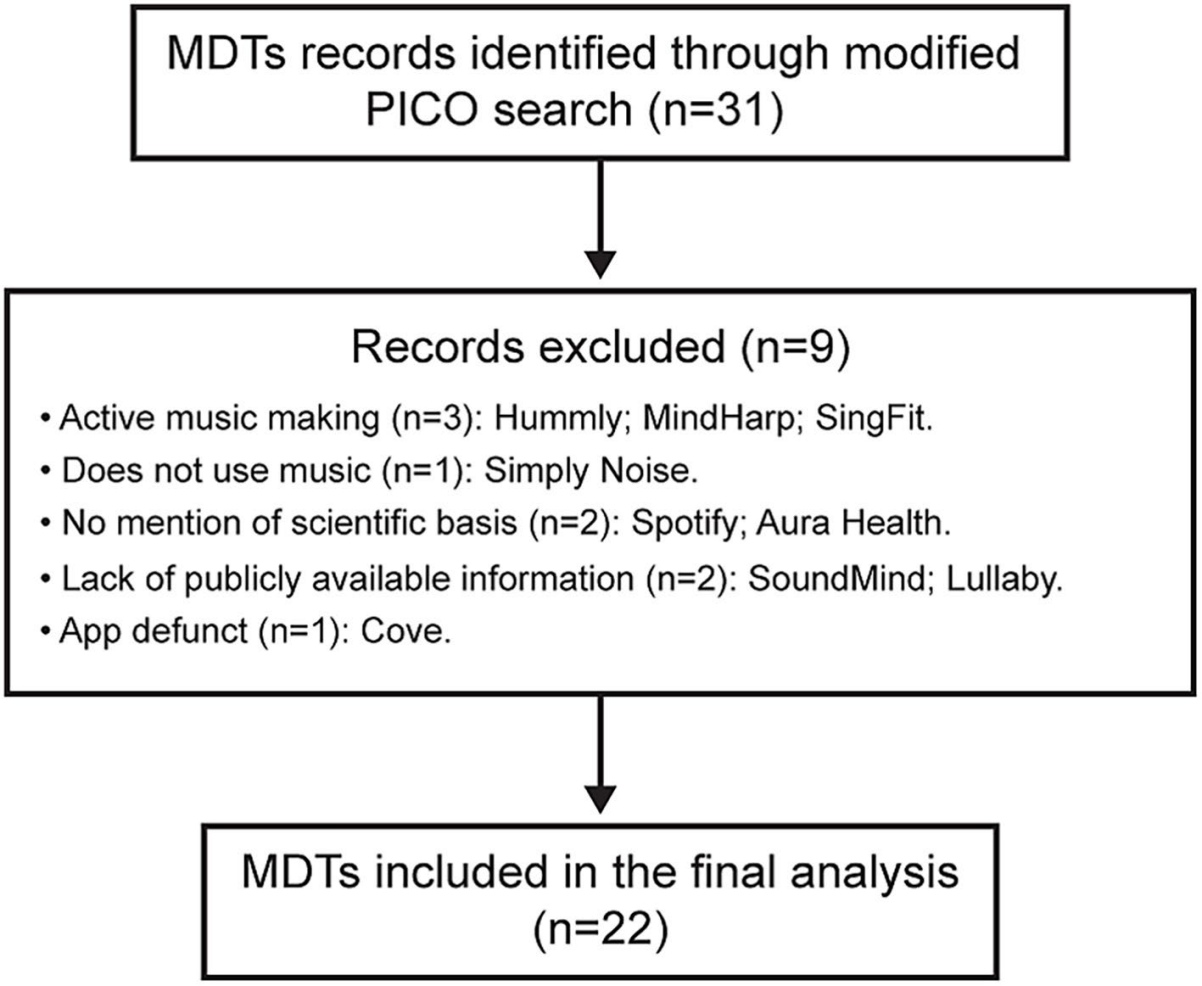
Flow diagram of music-based digital therapeutic identification and inclusion. PICO = population, intervention, comparison, outcome. MDT = music-based digital therapeutic.

Data collection was limited to publicly available materials, including company websites, blogs, and peer-reviewed publications. Information derived from third-party press coverage or media articles was excluded. This approach minimizes bias introduced by selective company participation. A flow diagram depicting the search and criteria application is shown in Figure 1. Limitations of this methodology are explicitly addressed in the Discussion.

## Results

A flow diagram documenting the search process is shown in Figure 1. Starting from a sample of 31 MDTs identified using the modified PICO search, a final list of 22 MDTs, each offered by a different company, were included for evaluation. A summary description of each of these MDTs is provided in Table 1.

**Table 1:** Summary of identified music-based digital therapeutics.

MDT	Advertised purpose	Summary	Target population	Music is primary intervention?	Published peer-reviewed empirical studies?
AlphaBeats	Focus, mood, performance (sports), relaxation	EEG headset-based neurofeedback using music* as feedback signal	General population	Yes	Yes
Brain.fm	Focus, relaxation, sleep	Ambient music composed by musicians including amplitude modulation at different EEG frequencies.	General population	Yes	Yes
BetterSleep	Relaxation, sleep	Curated music, soundscapes, and guided meditations	General population	No	No
Calm	Meditation, sleep, stress	Curated music, soundscapes, sleep stories, and guided meditations.	General population	No	Yes
Endel	Focus, relaxation, sleep	AI-generated soundscapes	General population	Yes	Yes
Feed.fm	Focus, Performance (sports), Relaxation	Curation music playlists for fitness and wellness	General population	Yes	No
Flow: Music therapy	Anxiety, emotional regulation, social and communication skills, speech and language skills	Platform for booking music therapy sessions	Autism spectrum disorders, attention deficit/hyperactivity disorder, pervasive developmental disorder, anxiety, depression, Alzheimer’s disease and dementia, sensory and perception disorders	Yes	No
Headspace	Mood, sleep, stress	Guided meditations, coaching, and curated music playlists	General population	No	Yes
HealthTunes	Anxiety, chemotherapy support, pain	Music composed with embedded binaural beats and isochronic tones	Clinical populations	Yes	No
InsightTimer	Anxiety, mood, sleep, stress	Guided meditations, curated music playlists, and soundscapes	General population	No	No
LUCID	Agitation, anxiety, Alzheimer’s, burnout	Music composed by musicians with the guidance from neuroscientists and music therapists; AI used to personalize playlists based on the Iso Principle for mood modulation from music therapy	Clinical populations: anxious disorders, Alzheimer’s disease, dementia	Yes	Yes
MediMusic	Anxiety, dementia, pain	Curated music playlists based on demographic data and listening preference data	Clinical populations: Alzheimer’s disease, dementia, peri-operative patients	Yes	No
MusicCare	Anxiety, mood, pain, sleep	Clinician-administered music composed by musicians with guidance from neuroscientists and music therapists; based on “U-sequence” for pain management	Clinical populations: anxiety disorders, depressive disorders, sleep disorders, peri-operative patients	Yes	Yes
Myndstream	Relaxation	Music composed and/or curated for spas	General population	Yes	No
Restful	Focus, over-stimulation, sleep	Curated ambient music embedded with binaural beats	General population, neuro-divergent populations	Yes	No
Sines App	Anxiety, depression, fatigue, focus, pain, performance (Cognitive), sleep	Music* with embedded binaural beats and guided meditations	General population	Yes	No
soundBrilliance	Calm, motivate, comfort, recover, pain, stress, energy, performance, wellbeing	“Enhanced” music composed by musicians. Audio paired with video of natural scenes to create audio-visual experiences.	General population	Yes	No
Spiritune	Anxiety, focus, mood, stress, sleep	Music composed by musicians based on parameters set by music therapists and neuroscientists for emotional transitions based on self-reported current and target emotional state, in different contexts	General population	Yes	Yes
SpokeWorld	Anxiety, focus, sleep	Curated music and lyrics based on the current and target state of user, and music preferences	General population	Yes	No
Thrive Global	Stress	60 s video clips of natural landscapes accompanied by curated music and guided breathing exercises	General population	No	No
Vera	Preference	Curated music playlists based on patient demographic information (e.g., place of birth, geographic location between ages of 15–35, etc.), entered into the app by caregiver.	Patients with Alzheimer’s disease or other dementia and their caregivers	Yes	No
Wavepaths	Anxiety, mood, hemodynamic response	Playlists of ambient music and soundscapes with interactive features to adjust emotionality, intensity, instrumentation, and therapeutic function	General population	Yes	No

EEG = Electroencephalographic; AI = Artificial Intelligence. *Whether music was composed or curated for app not stated in publicly available sources.

### Evaluation of published peer-reviewed empirical research associated with specific MDTs

Among the 22 MDTs listed in Table 1, 8 emphasize associations with published peer-reviewed empirical research. Of these, 6 MDTs were associated with research testing their effects on stress, anxiety, and/or mood (research related to BrainFM and Endel focused instead on attention). Of these, 4 MDTs were associated with research that explicitly manipulated music, permitting experimental evaluation of musical effects (research related to Headspace and Calm did not isolate music as an experimental factor, which is only one component of their meditation-based intervention). Inclusion of papers in Table 2 was limited to English-language, peer-reviewed, empirical publications. Music Cares has multiple publications in French which were not included. Furthermore, LUCID has published a perspectives article and several whitepapers which did not meet the inclusion criteria. Key details for each of the studies associated with these 4 MDTs are presented in Table 2.

**Table 2:** Summary of relevant published peer-reviewed empirical research.

MDT	Study	Population	*N*	Study design	Treatment	Control	Outcome
AlphaBeats	van Boxtel et al. (2012)	General population	50	Three-arm, between-subjects study	15 training sessions over 4 weeks; each session comprised three 8-min blocks of alpha neurofeedback training.	Beta training; No training	No significant changes in self-reported stress or relaxation, despite significantly increased alpha power in the treatment group.
	Dekker et al. (2014)	Athletes	13	Two-arm, between-subjects study	10 training sessions with variable durations over 4 weeks; each session comprised three 8-min blocks of alpha neurofeedback training	Beta training	No significant changes in self-reported stress or mood between groups.
	van Boxtel et al. (2024)	Athletes	41	Two-arm, within-subjects study	20 training sessions over 4–6 weeks; each session comprised four 5-min blocks of alpha neurofeedback training	No training	No significant effect of neurofeedback on self-reported mood.
LUCID	Mallik and Russo (2022)	Individuals with moderate to high trait anxiety currently taking anxiolytics	163	Four-arm, between-subjects study	Single session; session comprised 24-min of auditory beats with music	Music alone; Auditory beats alone; Pink noise	Significant reduction in self-reported somatic state anxiety compared to beats-alone and music-alone conditions in individuals with moderate trait anxiety. Significantly greater reduction in self-reported negative affect in music+beats condition compared to beats-alone in high trait anxiety individuals.
MusicCare	Guétin et al. (2009)	Patients with a chronic condition	30	Two-arm, between- subjects study	24 sessions of music therapy with one session per week over 24 weeks; each session comprised a 20-min block of a personalized “U-sequence” music intervention	Reading sessions	Significant improvements in self-reported anxiety and depression in music therapy group compared to reading session group.
	Guétin et al. (2009)	Patients with a chronic condition	13	Observational cohort	20 sessions of music therapy with one session per week over 20 weeks; each session comprised a 30-min block of a receptive music therapy based on the personalized “U-sequence” music intervention and a 30-min block of active music therapy based on playing an instrument	None	Significant improvements in self-reported mood between pre and post music therapy session. Significant reduction in self-reported anxiety-depression from week 10 to week 15 (for depression) and week 20 (for anxiety).
	Guétin et al. (2012)	Patients with a chronic condition	87	Two-arm, between-subjects study	At least 2 sessions per day for at least 8 days; each session comprised one 20-min block of a personalized “U-sequence” music intervention	Care as usual	Significant reduction in self-reported anxiety/depression, and anxiolytic consumption compared to control.
	Guétin et al. (2016)	Periprocedural patients	35	Observational cohort	Single session; session comprised one 20-min block of a personalized ‘U-sequence” music intervention	None	Reduction in self-reported anxiety between before and after session.
	Guétin et al. (2016)	Patients with a chronic condition	53	Observational cohort	Variable number of sessions (mean 7, up to 16); each session comprised one 20-min block of a personalized “U-sequence” music intervention	None	Reduction in self-reported anxiety between before and after session.
	Guétin et al. (2018)	Peripartum patients	62	Observational cohort	At least one session; each session comprised one 20-min block of a personalized “U-sequence” music intervention	None	Reduction in self-reported anxiety between before and after session.
	Messika et al. (2019)	Patients with a chronic condition	113	Three-arm, between-subjects study	Single session; session comprised one 30-min block “L-type” music intervention	Sensory deprivation/ Care as usual	Significant reduction in self-reported anxiety and depression in musical intervention group compared to sensory deprivation and care as usual groups upon ICU discharge. No significant differences between groups at 90-day follow-up.
	Demirtas et al. (2020)	Patients with a chronic condition	50	Two-arm, between-subjects study	Single session; session comprised one 20-min block a personalized “U-sequence” music intervention	Care as usual	Decrease in self-reported anxiety in both music intervention and control groups, but no significant difference between groups.
	Guerrier et al. (2020)	Periprocedural patients	309	Two-arm between-subjects study	Single session; session comprised one 20-min block of a personalized “relaxing musical program”	Silence	Significantly lower post-intervention self-reported anxiety in music group compared to control. Significantly lower hypertension related to anxiety post-intervention in music group compared to control.
	Guerrier et al. (2021)	Periprocedural patients	243	Two-arm, between-subjects study	Single session; session comprised one 20-min block of a personalized music intervention	Silence	No significant change in heart rate between the music intervention and control groups. Significantly lower self-reported anxiety post-intervention in the music intervention group compared to control.
	Parlongue et al. (2021)	Patients with a chronic condition	20	Single-arm trial	1–2 sessions per day for 3 months; each session comprised one 20-min block of a personalized “U-sequence” music intervention	None	Decrease in self-reported anxiety and depression from before to after listening.
	Bertacco et al. (2022)	Periprocedural patients	24	Two-arm, between-subjects study	Single session; session comprised one 40-min block of music intervention	Audiobook	No significant decrease in self-reported anxiety or change in negative emotions in music intervention compared to control group.
	Soyeux and Marchand (2023)	General population	28	Three-arm, within-subjects study	Single session; session comprised one 20-min block of a personalized “U-sequence” music intervention	Most-liked music, Least liked music	Significant decrease in self-reported anxiety in conditioned pain modulation condition compared to most-liked and least-liked music conditions. No significant difference in self-reported mood between the music-intervention group and control.
	Charron et al. (2025)	Periprocedural patients	170	Two-arm, between-subjects study	Single session; session comprised one 5-10-min block of a personalized “U-sequence” music intervention	Care as usual	Significantly greater decrease in self-reported anxiety between before and suturing process in music group compared to control. No significant change in HRV between groups.
Spiritune	Orpella et al. (2025)	General population	196	Four-arm, between-subjects study	Single session; session comprised one 10-min block of “work flow” music	Pop music/deep focus music/office noise	Significant increased, change in self-reported positive affect in WorkFlow condition compared to the pop music, deep focus, and office noise conditions.

Among the 14 MDTs for which no association with published peer-reviewed empirical research was found, Sound Brilliance, MediMusic, and Myndstream advertised results from experiments on their websites that had not yet been published, Wavepaths had published a prospectus article, Vera was associated with published literature reviews, and Feed.fm was associated with white papers that were neither empirical research nor peer-reviewed publications. The remaining nine MDTs did not assert an association or intent to conduct scientific research in the publicly accessible materials examined here.

**Figure 2:**
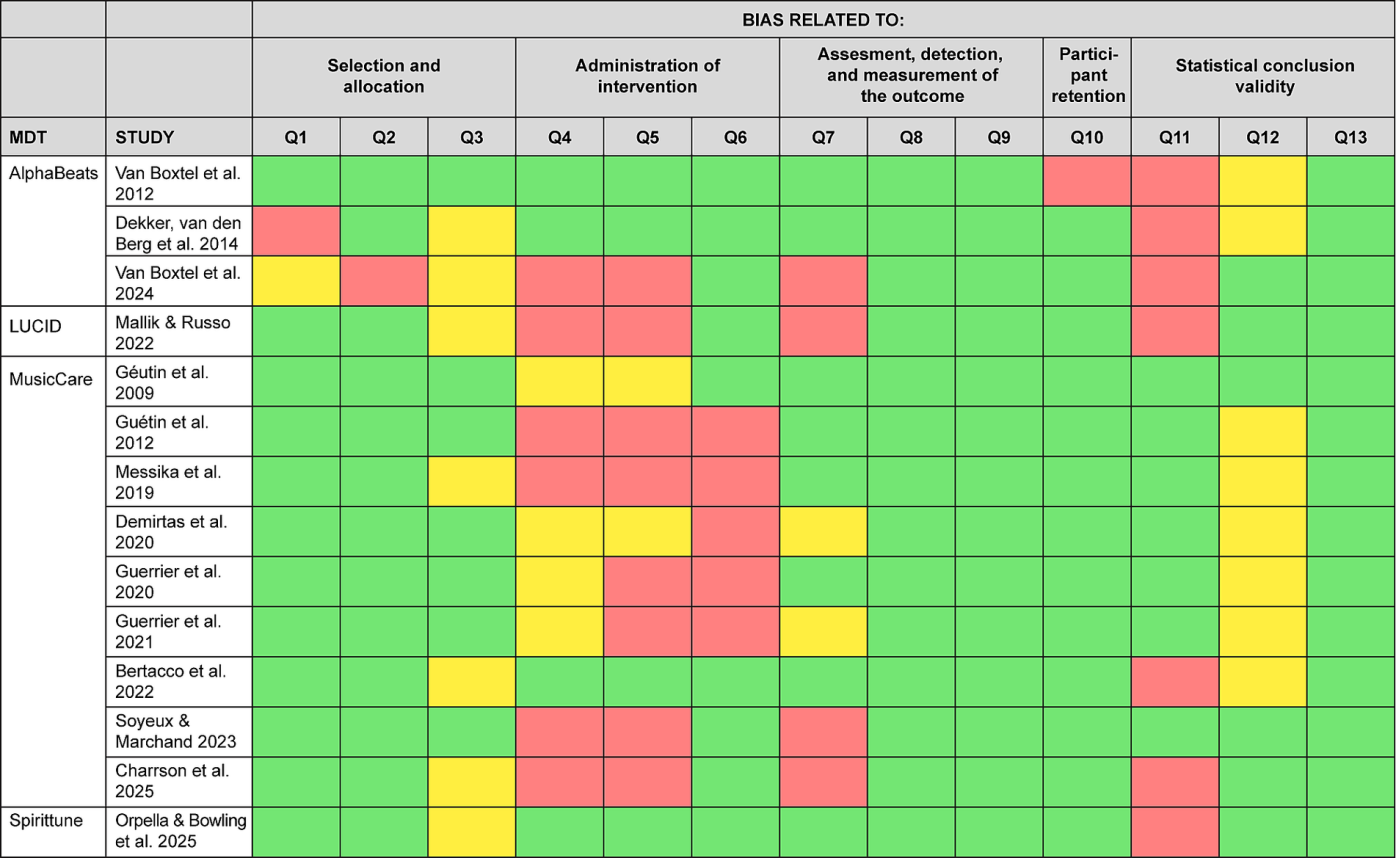
Risk of bias assessment for randomized controlled studies of MDTs. The following questions from JBI tool were evaluated with respect to stress, anxiety, and/or mood measurements. Q1. Was true randomization used for assignment of participants to treatment groups? Q2. Was allocation to treatment groups concealed? Q3. Were treatment groups similar at the baseline? Q4. Were participants blind to treatment assignment? Q5. Were those delivering the treatment blind to treatment assignment? Q6. Were treatment groups treated identically other than the intervention of interest? Q7. Were outcome assessors blind to treatment assignment? Q8. Were outcomes measured in the same way for treatment groups? Q9. Were outcomes measured in a reliable way? Q10. Was follow-up complete, and if not, were differences between groups in terms of their follow-up adequately described and analyzed? Q11. Were participants analyzed in the groups to which they were randomized? Q12. Was appropriate statistical analysis used? Q13. Was the trial design appropriate and any deviations from the standard RCT design (individual randomization, parallel groups) accounted for in the conduct and analysis of the trial?

Table 2 identified a total of 19 empirical papers, of which one was a single-arm trial (5%), four were observational studies (21%), and the remaining 14 were randomized controlled studies (74%). The methodological quality of these randomized controlled studies was assessed using the revised Joanna Briggs Institute (JBI) critical appraisal tool for randomized controlled trial, which comprises 13 items designed to systematically evaluate risk of bias (Barker et al., 2023). Each study was independently assessed by TV and AH, which responses recorded as yes, no, or uncertain for each item. All assessments were subsequently reviewed by DB, and any discrepancies were resolved by consensus through discussion and additional review. The results of the risk-of-bias assessment are summarized in Figure 2, with full justifications for each appraisal decision provided in the Supplementary Table 1.

### Major treatment strategies in MDTs

For clarity, the MDTs included in this review (see Table 1) are grouped into five categories based on their underlying treatment strategies, which are summarized in Table 3 and described in detail below. Importantly, this classification does not evaluate the effectiveness of individual MDTs for stress, anxiety, or depression, but rather maps the range of therapeutic approaches represented in the MDT landscape at the time of study.

**Table 3:** Employed by music-based digital therapeutics.

Treatment strategy	Definition	Example companies/platforms
Preference-based music selection	Curation or composition of music based on individual listener preferences	Calm, MediMusic, Thrive Global, Vera
Affective parameterization	Curation or composition of music based on featural associations with affect	Feed.fm, LUCID, Myndstream, Spiritune
Affect sequencing	Systematically varying musical affect over time to facilitate mood matching and change	LUCID, Music Care, Spiritune, SpokeWorld
Neural entrainment	Applying acoustic energy at specific frequencies in music to enhance corresponding neural oscillations	BrainFM, Endel, HealthTunes, LUCID, Restful, SinesApp, SoundBrilliance
Biofeedback	Using data from wearable biosensors to select or modify musical treatment content	Alpha beats, Endel, MediMusic, SoundBrilliance

#### Preference-based music selection

In neuroimaging research on musical reward, the engagement of brain affect and reward networks has primarily been studied by drawing contrasts between responses to preferred versus non-preferred music. Preferred music—defined as music or a musical style that a person likes more than others—reliably elicits greater activation, implying that musical enjoyment plays a key role in shaping the therapeutic effects of MDTs (and MBIs more broadly). Accordingly, tailoring MDTs to an individual’s music preferences may be essential for optimizing effects on stress, anxiety, and mood (Davis and Thaut, 1989; Jiang et al., 2013, 2016; Kim et al., 2003; Vella et al., 2024; Bowling, 2023). This may be especially important for individuals with relatively rigid or narrowly defined music preferences. Underscoring this point, a recent study compared the emotional impact of heavy metal and calm classical music in a sample of 90 young adults. While heavy metal generally increased negative affect, it had the opposite effect on a subset of individuals with a specific preference for it, increasing positive emotions such as joviality and serenity (Vella et al., 2024).

Recognizing the role of preference in shaping emotional outcomes, many MDTs incorporate personalized music selection into their design. The Vera app, for example, combines biographical information—such as birth year, place of birth, first language, and locations lived between ages 15–35—with metadata tagging and machine learning to generate individualized playlists for people with dementia. In a randomized controlled trial, Särkämö et al. (2014) used Vera as an intervention for patients with early-stage dementia, finding that 10 weeks of music listening was as effective as singing—and more effective than treatment-as-usual—for improving mood, orientation, and episodic memory. MediMusic employs a similar biographical approach to generate personalized playlists for improving mental health in dementia and Alzheimer’s patients, although the specific curation methodology is not publicly disclosed.

While some MDTs employ original compositions, AI-generated content, or production music deigned for broad appeal but unfamiliar to most listeners, others—such as Calm, Thrive Global, and soundBrilliance—incorporate commercially successful music by artists like Sam Smith and Ariana Grande. This strategy allows some users to engage with familiar, preferred music, which may increase therapeutic efficacy by more effectively stimulating brain reward and affect networks. However, this approach is not without risk: familiar, preferred music does not necessarily align with therapeutic efficacy, particularly for individuals whose preferences align with music that may reinforce negative mood states (Larwood and Dingle, 2022), or evoke distressing autobiographical memories (Sakka and Saarikallio, 2020). That said, if the benefits of familiar, preferred music outweigh potential risks, access to personally meaningful music may be central to future MDTs. This presents a problem in that many MDT developers lack the scale needed to navigate existing licensing frameworks, potentially giving large music streaming platforms with extensive catalogs and personalization systems a structural advantage.

A final consideration concerns the rapid rise of large language models, which are increasingly capable of generating music from text prompts linked to both stylistic preferences and therapeutic goals (Hou, 2022; Hu et al., 2020). MDTs may be particularly well positioned to integrate such tools, but AI-based music generation raises significant ethical challenges. These include the uncredited or exploitative use of creative works as training data, potentially undermining the livelihoods of musicians whose work informs these models; the further erosion of social and interpersonal dimensions already diminished in MDTs relative to therapist-led MBIs; and trust-based concerns about emotional manipulation through AI— even when pursued for therapeutic purposes. Whether these issues will ultimately affect the therapeutic value of MDTs that incorporate AI-generated music remains unclear, but they warrant careful and ongoing ethical consideration as these systems evolve.

#### Affective parameterization

While personal preferences undoubtedly influence the psychological and physiological effects of music, certain musical features—such as tempo, mode, and timbre—exert characteristic effects on affect (Dickson and Schubert, 2022; Elliott et al., 2011; van der Zwaag et al., 2011). The relationship between these features and emotional expression is broadly conserved across both speech and music (Juslin and Laukka, 2004; Weninger et al., 2013) and, to some extent, even in animal communication (Briefer, 2012; Filippi et al., 2017). This cross-domain consistency likely reflects the constraining influence of affective physiology on biological sound production (Bowling, 2023).

Some of the most robust associations between musical features and affect include those between tempo and perceived energy (Dickson and Schubert, 2022; Xue and Wang, 2022), spectral energy distribution and arousal (Bowling et al., 2019; Tsai et al., 2010; Arnal et al., 2015), and consonance and positive affect (Bowling et al., 2012; Bowling and Purves, 2015; Bowling et al., 2017). These and related feature-affect relationships provide a biological framework for quantifying musical affect—at least as a first approximation—recognizing that emotional significance will vary across individuals due to differences in learning, memory, and contextual interpretation. Although current curation efforts for therapeutic music are primarily being implemented subjectively (i.e., not guided by explicit bioacoustic cirteria), objective methods for feature extraction and emotional classification are advancing rapidly (Agres et al., 2021; Volk et al., 2023; Kang and Herremans, 2025). By modeling these feature–affect relationships, MDTs can systematically select, sequence, or generate music to target specific emotional states, toward a more precise and replicable basis for affective modulation.

MDTs integrating affective parameterization on the compositional side design music with predefined emotional intent. One example comes from Spiritune, which produces music created by composers working in collaboration with neuroscientists and music therapists to stimulate positive mood across different contexts. In a 2025 study, Orpella, Bowling, and colleagues randomized 196 adults into one of four groups, each exposed to a different auditory condition for 10 min while performing a cognitive task (a combined flanker and no-go paradigm). Participants who listened to Spiritune’s “work flow” music—parameterized to support a transition from anxious to energized states—showed significantly greater mood improvement, reflected in increased positive affect and reduced negative affect, compared to those who listened to contemporary popular hits, a widely used “deep focus” playlist, or ambient office noise (negative control). Additionally, only the work flow group demonstrated enhanced cognitive performance, with reaction times improving over the course of the task, while participants in all other groups showed progressive slowing. Notably, these effects were consistent across a range of baseline mental health levels, as assessed by a standardized screener for depression, anxiety, and stress (Orpella et al., 2025). These findings highlight the potential of affective parameterization in MDTs to enhance emotional and cognitive outcomes.

As with preference-based treatment strategies, MDTs may offer specific advantages for affective parameterization. In traditional music therapy, clinicians curate or create music within a therapeutic relationship to address emotional or behavioral needs, dynamically adjusting parameters such as tempo, melody, and harmony in real time or across sessions (Wheeler, 2015; Aalbers et al., 2017). This individualized process—guided by clinical training, intuition, and interpersonal attunement—remains central to music therapy, enabling nuanced emotional responsiveness that is difficult to reproduce technologically. However, therapists are naturally constrained by the breadth of their repertoire, the features they can monitor and modify, and the generalizability of their experience-based heuristics across listeners. MDTs have the potential to overcome many of these limitations by integrating information about individual preferences with large, acoustically annotated music libraries characterized by affective features (Dickson and Schubert, 2022). With continued advances in sensitivity to listener affect—via analyses of facial expression, movement, or physiological signals—MDTs may increasingly be able to model the relationships between acoustic features, individual responses, and therapeutic outcomes. This could enable scalable affective parameterization that preserves key aspects of the real-time emotional attunement characteristic of interaction with a skilled music therapist.

#### Affect sequencing

Closely related to affective parameterization is affect sequencing. Across many forms of music, affect is intentionally structured to guide listeners on an emotional journey—a principle evident in the arrangement of concert programs, symphonies, albums, and individual songs. While such sequences are typically crafted based on artistic intuition, they can also be systematically designed by selecting and arranging musical pieces according to the affective properties of their musical features (Davis, 2003). In music therapy, affect sequencing is formalized in the Iso Principle, a mood modulation technique in which therapeutic music is first matched to a patient’s current mood state and then gradually transitioned toward music that evokes a desired mood state (Altshuler, 1944). First described in the context of in-patient care for WWII veterans, the Iso Principle remains one of the few core practices consistently used across diverse music therapy approaches (Davis, 2003; Heiderscheit and Madson, 2015; Wigram et al., 2002). Recent evidence indicates that the Iso Principle can modulate mood in as little as 5 min. Starcke and von Georgi (2024) found that experimentally induced self-reported sadness was alleviated more effectively when participants listened to a sad song followed by a happy one, compared with two consecutive happy songs (Starcke and von Georgi, 2024). This suggests that meeting listeners “where they are” emotionally before attempting to shift their mood is an important part of successful mood modulation. It further suggests that affect sequencies can be effectively applied in playlists of recorded music deliverable through MDTs.

At least two of the MDTs reviewed here—Spiritune and LUCID—explicitly incorporate the Iso Principle into their music programming. Spiritune does so by prompting users with two questions before track selection—*“How are you feeling?”* and *“How do you want to feel?”*—and then selecting music from its catalogue that matches the desired emotional trajectory. LUCID employs a similar approach, allowing users to indicate their current emotional state either by taking a photo of their face (used to assess arousal and valence) or by selecting a position on a visual tool loosely based on the Russell Circumplex model of affect. After listening, users can track changes in their mood using the same tool. However, the specific methods used by these platforms to match and sequence tracks remains undisclosed, leaving open questions about personalization as well as the algorithms underlying their interventions.

A related concept, the U-shaped Curve Technique, applies affect sequencing to the management of anxiety and pain. Developed in the context of hypnoanalgesia research at the University Hospital of Montpellier, this approach—proprietary to MusicCare—is based on the premise that patient-preferred music can serve as a distraction from anxiety and pain. MusicCare’s method involves structured musical sequences in which tempo and orchestration density begin at a moderate level, gradually decrease into a calm “valley” for a defined period, and then rise again to their original levels, forming a characteristic U-shaped arc (Guétin et al., 2014). Like Spiritune, MusicCare uses original music composed by musicians in collaboration with neuroscientists and music therapists. MusicCare stands out for having conducted multiple validation studies in clinical settings, with findings indicating moderate-to-large effects in reducing self-reported anxiety. For example, in a study of patients with chronic back pain, four U-shaped MusicCare treatments delivered alongside standard care produced large pre-to-post reductions in anxiety (d = 0.80) relative to standard care alone (Guétin et al., 2005). Similarly, in the cataract surgery, two separate studies reported greater anxiety reduction among patients receiving a single 20-min U-shaped MusicCare session compared with silent control conditions (Guerrier et al., 2021).

While these findings highlight the promise of structured affect sequencing in MDTs—not only for mood modulation but also for anxiety and pain management—the contribution of specific emotional/musical trajectories (e.g., unidirectional or U-shaped) has yet to be empirically isolated. This would require direct comparison of music organized in trajectories hypothesized to be therapeutic with music organized in an alternative or inverse trajectory.

#### Neural entrainment

EEG research shows that musical rhythm can elicit sustained frequency-following responses, selectively enhancing phase-locked neural oscillations at frequencies corresponding to the musical pulse (or “beat”) and its nested rhythmic cycles (Doelling and Poeppel, 2015; Harding et al., 2019; Nozaradan et al., 2011; Trost et al., 2014). These findings have led to the hypothesis that music can be designed to enhance activity within specific frequency ranges through processes of synchronization between systems through interaction and/or enhanced response at a system’s natural frequency, thereby amplifying endogenous neural oscillations to boost associated brain functions.

In this context, different frequency bands within the spectrum of EEG-detectable neural oscillations are typically associated with different cognitive and physiological states (e.g., Aarts et al., 2014; Carvajal et al., 2024; Stupacher et al., 2016). For example, delta waves (0.5–4 Hz) are linked to deep sleep, homeostatic regulation, and unconscious states (Borbély, 2022; Knyazev, 2012). Theta waves (4–8 Hz) are associated with light sleep, meditative or trance-like states, deep relaxation, and memory encoding and retrieval (Baijal and Srinivasan, 2010; Hebert and Lehmann, 1977; Kahana et al., 2001). Alpha waves (8–12 Hz) are associated with relaxed wakefulness, calm focus, and reduced sensory input (e.g., eyes closed; Klimesch, 1999; Marshall and Bentler, 1976). Beta waves (12–30 Hz) are associated with active thinking, problem-solving, sustained attention, and anxiety (Kamiński et al., 2012; Roxburgh et al., 2023; Spitzer and Haegens, 2017). Gamma waves (30–100 Hz) are associated with high-level information processing, sensory integration, attention, memory, and most recently, reductions in amyloid-*β* and Tau pathology in Alzhiemer’s Disease (Ichim et al., 2024; Kaiser and Lutzenberger, 2003; Tallon-Baudry and Bertrand, 1999). However, oscillations within each of these frequency bands likely arise from multiple circuits distributed across the brain, reflecting diverse and context-dependent functions that may not align with the premise that frequency-seeded music can globally drive brain activity in a desired therapeutic direction. Thus, while frequency-based modulation in music may systematically influence neural dynamics, the extent to which it can reliably enhance specific cognitive or affective states remains unclear.

Despite these complexities, neural entrainment remains a fundamental aspect of music perception, and several MDTs leverage this principle. One widely studied approach involves auditory beats, a form of amplitude modulation produced by the interaction of tones with different fundamental frequencies (e.g., separated by approximately 2–40 Hz). A growing body of evidence indicates that auditory beats can influence cognitive and affective states (Chaieb et al., 2015; Draganova et al., 2008; Garcia-Argibay et al., 2019; Ingendoh et al., 2023; Padmanabhan et al., 2005; Isik et al., 2017). Auditory beats can be generated in two primary ways. Monaural beats arise when the interaction between tones occurs before the sound reaches the ear, whereas binaural beats arise when separate tones are presented to each ear, producing the interaction centrally within the brain, presumably through spatial hearing mechanisms (Chaieb et al., 2015; Schwarz and Taylor, 2005; Oster, 1973). Although monaural beats generally elicit stronger neural responses (Schwarz and Taylor, 2005), most research on stress and anxiety-reducing effects has focused on binaural beats. Despite considerable variability in methods for producing and evaluating binaural beats, a recent meta-analysis found that binaural beats in the theta/delta frequency range have a medium-to-large effect on anxiety reduction (Hedges’ *g* = 0.69, based on five effects sizes in four studies; total *N* = 159; Garcia-Argibay et al., 2019). While Garcia-Argibay et al. (2019) suggests that embedding auditory beats in music might reduce their efficacy due to potential conflicts between the rhythmic beat of the music and the beating frequency of the auditory beat, recent research has described methods for integrating monaural beats matched to the harmony and rhythm of source music, without perceptual or emotional disturbance (Venkatesan et al., 2025).

LUCID integrates auditory beats within their music to facilitate neural entrainment and relaxation. In a controlled study, Mallik and Russo (2022) compared LUCID’s *“relaxing”* music—embedded with theta-frequency auditory beats—with three other conditions: music without beats, auditory beat stimulation alone, and a pink noise control. Participants were individuals with moderate-to-high anxiety who were currently taking anxiolytic medication. Results indicated that the LUCID condition produced greater reductions in both cognitive (e.g., difficulty concentrating) and somatic (e.g., sweating) aspects of self-reported state anxiety compared with all other conditions (Mallik and Russo, 2022).

Another approach to neural entrainment involves amplitude modulation, wherein loudness fluctuations are introduced at specific frequencies. Research suggests that this type of modulation can induce neural entrainment effects (Doelling and Poeppel, 2015; Joris et al., 2004; Woods et al., 2024). One company applying this technique is Brain.fm, which adds amplitude modulation at different frequencies to music designed for focus, relaxation, and sleep. In a recent peer-reviewed study, Woods et al. (2024) found that music with beta amplitude modulation (peaking around 14–16 Hz) elicited greater activity in key nodes of the brain’s salience network (measured with fMRI) and stronger neural entrainment (measured with EEG) compared with music containing slower, less-focused modulation in the 0–8 Hz range. Behaviorally, participants with higher scores on the Adult ADHD Self-Report Scale (reflecting inattention, impulsivity, and hyperactivity) showed improved performance over time on a sustained attention task when listening to beta-modulated music, relative to the same music with slower or faster added amplitude modulation (8 Hz or 32 Hz, respectively; Woods et al., 2024). Although the study did not directly examine the effects slower delta and theta modulations on stress or anxiety reduction, these findings suggest that analogous entrainment mechanisms could be leveraged for relaxation-based interventions, similar to those observed with auditory beats.

From an MDT perspective, incorporating amplitude modulation or auditory beats into music is relatively straightforward, as these signals can be software-generated and easily integrated with digital recordings. Machine learning algorithms can be used to analyze the harmonic and rhythmic content of individual music tracks and integrate sympathetic beat frequencies or amplitude modulation (Venkatesan et al., 2025), potentially increasing efficacy. While such strategies could, in principle, be applied within traditional MBIs—for example, by introducing auditory beats to music during live therapy sessions—this approach to boosting frequency-specific neural entrainment has not historically been part of therapist-delivered interventions, though it is often implemented in sound “bath” treatments (see Johnson, 2025).

#### Biofeedback

Music playback can serve as a powerful auditory cue for physiological feedback, allowing biosensor data to be translated into salient auditory signals. In such applications, physiological signals—such as heart rate or EEG spectra—are used to shape musical feedback, either through real-time composition (e.g., “sonification”; Kramer et al., 2010) or by dynamically modifying pre-recorded music. Listeners can then use these musical changes as biofeedback cues to regulate their physiological state (e.g., lowering heart rate or increasing EEG power within a target frequency band), potentially influencing stress, anxiety, or mood.

Several studies have demonstrated the efficacy of music as a biofeedback signal (e.g., Liu et al., 2009). For example, a study by Bergstrom et al. (2014) implemented a system in which tempo and amplitude dynamically in response to the listener’s heart rate—accelerating and intensifying as heart rate increased, and slowing and softening as heart rate decreased. This form of music-based biofeedback promoted more effective self-regulation of physiological arousal than listening to a pre-recorded playlist and performing comparably to a non-musical sine-wave biofeedback signal that varied in pitch based on heart rate (Bergstrom et al., 2014).

AlphaBeats leverages EEG-based biofeedback to enhance alpha wave activity (8–12 Hz) through music. The system dynamically adjusts the cut-off frequency of a high-pass filter, allowing a listener’s preferred music to be heard more clearly when relative power in the alpha band (measured via alpha/beta ratio) is high, thereby reinforcing the desired brain state.

In a study by van Boxtel et al. (2012), 50 participants completed 15 24-min training sessions over 4 weeks, receiving either alpha biofeedback (targeting the 8–12 Hz range), beta biofeedback (focused on a 4 Hz bin within the 14–30 Hz range), or music playback without biofeedback. Post-intervention results showed that alpha training produced a significant 10% increase in alpha activity—an effect not observed in the beta or music-only groups. This increase persisted at a three-month follow-up, suggesting potential long-term neuroplastic effects. Participants in the alpha group were also more than twice as likely to describe the training as relaxing compared with those in the other groups. Although behavioral measures of stress, relaxation, and sleep also favored the alpha training group, these differences were not statistically significance (van Boxtel et al., 2012).

While AlphaBeats provides direct biofeedback, other platforms—such as Endel—use biometric and contextual data to shape real-time adaptive soundscapes. Rather than delivering explicit feedback for physiological self-regulation, Endel employs AI to generate personalized auditory environments based on a combination of physiological inputs (e.g., heart rate) and contextual cues (e.g., time of day, weather). In a study by Haruvi et al. (2022), Endel’s soundscapes were compared to silence and curated playlists from popular streaming platforms to assess their effects on “focus” during tasks such as playing Tetris, solving math problems, and completing word puzzles. Focus was assessed by self-report, as well as EEG measures (e.g., an “engagement ratio” defined by beta/[alpha + theta]). The results showed that the engagement ratio correlated with self-reported focus, with personalized soundscapes resulting in significantly larger ratios than silence. Curated playlists showed more variable effects. No significant differences were observed between conditions in self-reported stress or relaxation.

Together, these findings suggest that integrating music-based biofeedback into MDTs offers a promising route for enhancing physiological self-regulation and cognitive performance. Some platforms deliver real-time feedback, while others passively adapt auditory environments based on biometric or contextual inputs. Although such approaches may be uniquely well-suited to MDTs (as opposed to more traditional MDTs), their scalability is currently constrained by hardware. This is especially evident in brain-based biofeedback, where consumer-grade EEG systems—using far fewer channels than laboratory-grade systems—are more affordable and portable but also produce sparser, lower quality data with lower signal-to-noise and more artifacts (Peake et al., 2018; Sawangjai et al., 2019). Ongoing efforts to improve software, reduce costs, and validate performance are encouraging (Sabio et al., 2024), yet the trade-off between data quality and accessibility remains a central limitation to large-scale deployment.

## Discussion

Much of the translational appeal of music-based digital therapeutics (MDTs) lies in their potential to expand access to music-based treatment for stress, anxiety, and depression. Despite this promise, few published, peer-reviewed, empirical studies have tested whether MDTs deliver benefits comparable to—or distinct from—other forms of music-based intervention, or even from everyday music listening, particularly in real-world settings (but see Mallik and Russo, 2022; Orpella et al., 2025). Although the broader evidence supporting music-based interventions (MBIs) for reducing stress, anxiety, and depression is generally strong, it remains unclear whether MDTs specifically reproduce the therapeutic effects observed in more traditional, clinician-delivered music-based interventions, or whether their apparent benefits are driven largely by non-specific or contextual factors, as discussed below.

Currently, scientific validation of MDTs remains limited. Of the 20 companies identified in this review which claimed to be “backed by science” or “scientifically proven,” only eight have published peer-reviewed empirical research explicitly examining the effects of music, and only two (MusicCare and AlphaBeats) have published multiple studies. To our knowledge, only MusicCare and Alpha Beats have examined longitudinal outcomes with extended follow-up. LUCID and Spiritune have only published studies with single session exposure for 24-min and 10-min, respectively. The remaining 12 MDTs only advertised preliminary, unpublished results or secondary literature, which, in some cases, was not directly relevant to their principal treatment strategy.

The risk-of-bias assessment further highlights substantial methodological limitations across the literature: studies were frequently open-label, relied on waitlist or inactive (silent) control conditions, or failed to adequately describe blinding procedures. Notably, 31% of studies did not report participant blinding methods, and 15% did not report experimenter blinding. Moreover, six of the 20 studies employed single-arm or observational designs, precluding causal inference.

Evidence comparing MDT-specific treatment strategies to generic music listening is mixed. While some studies report advantages of specific MDT-based approaches over other forms of music (e.g., van Boxtel et al., 2012; Mallik and Russo, 2022; Haruvi et al., 2022; Orpella et al., 2025), others find comparable effects between conditions (e.g., Wiwatwongwana et al., 2016). Interpretation of these findings is further complicated by unclear or insufficient statistical reporting: over half of the randomized controlled studies reviewed here were rated as having “unclear” statistical analyses, with some relying solely on post-intervention or change-score comparisons without adequately accounting for baseline differences (see Supplementary Table 1, Q12). Given that music listening is already widely perceived as beneficial for mental health (Fink et al., 2021; Hatton, 2023), it remains unresolved whether MDTs offer distinct therapeutic advantages beyond more typical forms of engagement with music.

These scientific uncertainties are compounded by well-known challenges in behavioral medicine related to blinding, bias, and placebo effects. Double blinding is often difficult to achieve in trials of therapies that require active involvement from unblinded practitioners and investigators—like psychotherapy—potentially introducing differential treatment delivery. Unblinded participants may also expect to benefit or may unconsciously align their responses with perceived study goals, giving rise to expectancy and demand biases (Benedetti, 2021; Enck and Zipfel, 2019). These issues are particularly pronounced in MBI research, where it is inherently difficult to “hide” the intervention, as both participants and investigators typically know whether and when a musical intervention is being delivered. An additional source of placebo effects arises from self-selection. Individuals who choose to engage with music-based interventions are likely to hold preexisting expectations of benefit, to appreciate the emotional power of music, and to have a favorable orientation toward behavioral (i.e., non-pharmacological) approaches to health.

At the same time, several features of MDTs may help mitigate some of these biases. Remote delivery and pre-programmed protocols can reduce direct investigator involvement and may be more easily configured to limit participants’ awareness of alternative conditions. As MDTs become increasingly standardized, it may become possible to evaluate the real-world benefits of specific musical treatment strategies using protocols that are identical in all respects except for the treatment component under investigation—e.g., music with auditory beats versus music without auditory beats [see Mallik and Russo, 2022 for a similar but laboratory-style open-label study]—providing a clearer path toward a more rigorous and reproducible evidence base.

Isolating music-specific effects presents additional challenges. MDTs typically incorporate auxiliary features—such as mood check-ins, reminder notifications, and progress tracking—that can independently promote positive behaviors, including self-awareness, adherence, and goal-directed engagement, and can influence outcomes irrespective of music content (Bakker and Rickard, 2018; Chandrashekar, 2018; Huberty et al., 2021; Lee et al., 2018). These auxiliary features may amplify the “digital placebo” effect, which stem from positive expectations about digital tools, heightening users expectations of efficiency and benefit (Torous and Firth, 2016).

Moreover, music is frequently combined with other non-musical treatment components, as in multi-component wellness applications like Headspace and Calm that integrate music with meditation or breathwork. While such platforms often report stress-reducing effects (Champion et al., 2018; Stecher et al., 2021; Huberty et al., 2021, 2022), the specific contribution of music remains difficult to disentangle. MDTs may also combine multiple concurrent treatment strategies (e.g., biofeedback, affective parameterization), further complicating independent assessment of their individual contributions.

Together, these factors represent significant obstacles to isolating the music-specific mechanisms underlying MDTs. Importantly, however, this experimental challenge is partially decoupled from practical treatment considerations, where the intentional combination of features and strategies is often designed to maximize user engagement and therapeutic benefit. Nonetheless, these constraints limit scientific insight into music-specific mechanisms and how they might be optimized for therapeutic impact.

### Recommendations for sustainable MDT development

To address the challenges outlined by this review, we propose three recommendations to support the sustainable development MDTs toward fulfilling their promise of improving health and wellbeing at scale.

#### Back up claims of being “science-backed”

We recommend that MDT developers move beyond reliance on vague marketing terms such as “science-backed,” which are currently applied to such a broad range of evidence that they have limited meaning and may be misinterpreted, particularly by non-experts. We therefore recommend that such claims, when used, be accompanied by clear disclosure of the evidence being referenced, with MDT developers committing to transparent strategies for building more specific evidence over time. This should include well-controlled, double-blind laboratory studies testing specific treatment strategies, replication across independent samples, and ultimately randomized double-blind placebo-controlled trials conducted in ecologically valid settings to assess real world adherence and effectiveness.

Leveraging the scalability of MDTs to study large and diverse populations can further support refinement of dosage, identification of subgroup-specific effects (e.g., cultural or genre-based preferences), and clarification of who is most likely to benefit. An important component of this strategy is the use of outcome measures that can be assessed remotely via personal devices, aligning evaluation with how MDTs are delivered. These may include biological signals (e.g., photoplethysmography-based indices of cardiovascular function), psychological outcomes (self-reported stress, anxiety, and depression), and behavioral or social indicators (e.g., movement or location-based measures of time spent outside the home). Recognizing that early-stage MDT development typically occurs under resource constraints, this progression can proceed incrementally, with early rigor and transparency laying the foundation for later clinical trials.

#### Emphasizing the role of artistic insight

Developers should clearly distinguish between components of an MDT that are grounded in established scientific evidence and those guided by experiential, artistic, or practitioner-derived insight. The latter should not be treated as a weakness: musicians and music practitioners are often uniquely skilled at shaping music to produce reliable emotional, cognitive, and physiological effects, and their expertise represents a valuable source of innovation. However, such contributions should be described as artistically or experientially informed rather than framed as scientifically validated when experimental evidence is not yet available—a distinction too often blurred in MDT marketing. When communicated honestly, this distinction elevates both forms of insight, respecting the deep expertise gained through musical practice while underscoring the role of science in measuring, testing, and personalizing therapeutic applications.

#### Prioritize rigorous controls

Future MDT research should incorporate control conditions capable of isolating both music-specific and strategy-specific effects. These include *sham* music-based controls in which listening is prescribed but key therapeutic ingredients (e.g., biofeedback) are deliberately removed; active non-musical controls, such as meditation-based therapeutics delivered without music; and passive “*music-as-usual”* controls in which participants’ typical listening behavior is tracked for comparison. Together, these control conditions enable more precise differentiation between the effects of structured musical intervention, broader engagement with digital wellness tools, and everyday music use. The standardized, programmable, and scalable nature of MDTs makes such designs particularly feasible. Identical app interfaces can be used to deliver both intervention and control content, facilitating blinding and minimizing expectancy differences across conditions. Moreover, modular delivery allows individual components—such as personalization algorithms, auxiliary features, or acoustic parameters—to be selectively included or withheld, supporting mechanistic investigations into *what* aspects of MDTs drive therapeutic benefit and *why*.

#### Meaningful transparency

Across the above recommendations, MDTs should prioritize user-centered transparency regarding proposed therapeutic mechanisms and the evidence supporting them. As personalization strategies expand—particularly with the integration of AI-driven music generation or remixing—developers should provide clear, accessible explanations of what data are collected, how those data are used, and how interventions may or may not adapt in response. Such transparency is critical for shaping the acceptability of MDTs, defined as the extent to which individuals delivering and receiving them perceive the intervention as appropriate based on anticipated or experienced cognitive and emotional effects (Sekhon et al., 2017). Transparency is also essential for protecting data privacy, as the use of behavioral and biosensor data introduces risks related to the handling of personal health information. Together, these considerations are central to informed consent, ethical deployment, alignment of expectations with reality, and eventual medical integration.

These recommendations aim to support research and development practices that establish MDT efficacy and enable the sustained investment needed to scale their impact responsibly. Grounding innovation in transparent evidence-building and rigorous evaluation is essential for expanding music’s therapeutic potential to a level not previously achieved in modern contexts. Without such a foundation, rapid commercialization risks mischaracterizing this potential, echoing earlier examples—such as Muzak or the “Mozart effect”—in which promising findings were amplified beyond the supporting evidence, ultimately undermining public trust (Bowling, 2023; Lanza, 2004).

### Methodological limitations of this review

This review is subject to several methodological limitations. Most fundamentally, there is no centralized repository or standardized indexing system for companies developing MDTs, which precludes fully systematic identification of products and limits reproducibility. Although a modified PICO-based web search provided a pragmatic means of mapping this emerging field, the resulting coverage cannot be considered exhaustive.

The search was conducted only in English and may therefore have excluded non–English-language MDTs. In addition, comprehensive coverage is constrained by the rapidly evolving commercial landscape. Since the January 2025 search, several MDTs have updated their products or websites (e.g., Brain.fm, LUCID, Spiritune, SoundBrilliance, Wavepaths), at least one company has declared bankruptcy (AlphaBeats), another company website appears to have been taken down (Vera), and new offerings are under development (e.g., Sonocea, Oscillo Biosciences). Data collection was further limited to information available directly from MDT platforms (apps and websites), excluding third-party press coverage. While this choice reduced reliance on promotional or selectively reported claims, it likely limited insight into ongoing or unpublished research activities. For example, although not described on its website, a 2024 press release from Anglia Ruskin University indicates that MediMusic is conducting research on how AI technology and music can be used to ease anxiety symptoms in people of South Asian descent living with dementia (ARU Press Office, 2024). The release also mentions previously completed pilot clinical trials with Lancashire Teaching Hospitals NHS Foundation Trust, although there does not currently appear to be a record of whitepapers or publications based on these trials online.

Despite these limitations, the 22 MDTs identified in Table 1 provide a representative snapshot of prominent music-based therapeutic interventions promoted within the commercial wellness sector as of January 2025. To our knowledge, this is the first review to systematically map this space—allowing for a description of principal treatment strategies and an evaluation of supporting evidence and recommendations for further development. As MDTs continues to evolve, future reviews will have an important role to play in uniting the field, tracking progress and advancing music-based treatments for human health and wellbeing.
